# Dr. Sibson on the Means of Determining the Relative Position of the Thoracic and Abdominal Viscera

**Published:** 1844-01

**Authors:** 


*Dr. Sibson on the Means af Determining the Relative Position of the Thoracic and Abdominal Viscera.*—At the late anniversary meeting of the Provincial Medical and Surgical Association held at Leeds, Mr. Sibson stated, that in 1836, when examining patients with chest affections, he felt his knowledge of the relative position of the thoracic and abdominal viscera was imperfect and confused. To remedy this he took outline sketches of the viscera from most of the bodies that were dissected in the General Hospital, near Nottingham, at first by the eye, afterwards by the aid of a frame. After some time, Dr. Hodgkin, of London, suggested to Mr. Sibson a very valuable plan, facilitating and rendering more accurate the diagrams. This consisted in placing a frame over which a piece of net or lace was stretched, above the body the organs of which were to be sketched, and by tracing with chalk the outline of each organ, as seen through the gauze. Mr. Sibson then proceeded to give a rapid sketch of the general results obtained by the carrying out of this plan.

The apex of each lung ascends above the clavicle from one to two inches. This is an important practical fact, as by examination of this region tubercles may be detected in their early stage, and long before they have extended to that part of the lung below the clavicle. The inner line of the right lung is behind the sternum for its whole length, whereas that of the centre of left lung diverges obliquely outwards and downwards opposite to the fourth costal cartilage. The lower edge of the right lung is generally behind the sixth costal cartilage; that of the left lung is about a rib's breath lower. The upper margin of the liver, from the dulness of that organ, forms an admirable means for distinguishing the lower border of the resonant right lung. The hollow resonance of the stomach in contrast with the diffused resonance of the lung enables the lower border of the left lung to be defined with equal facility.

In emphysema the chest is constantly in the state of a very deep inspiration; the lower margin of the lung descends several ribs' breadth below its usual seat; at the same time the diaphragm draws down the lungs; it in a like manner and extent draws down the heart. The dilatation of the structure of the lungs is due not to frequent coughing, but to constant efforts, by extreme inflation of the lungs, to relieve dyspnœa, the structure of the lungs being in this manner overstretched.

In pneumonia, pleuritis, and general tuberculous infiltration of the whole lung, the affected organ is greatly enlarged, the lower margin descends considerably below its normal position, and the walls of the chest on the diseased side are much more expanded than on the healthy one, but the healthy lung expands more than the diseased one on a deep inspiration. In phthisis affecting the summit of the lung, the mass of the lungs does not appear to be much affected in size. In the consolidation and contraction of lung following pleuritis, the whole structure is diminished and the lower margins are raised, their previous position being occupied by the abdominal viscera.

On artificially distending the pericardial sac, it assumes the form of an oblate spheroid, on the top of which is placed a smaller hemisphere which surrounds the great vessels. In pericarditis with effusion, the sac assumes the same form and displaces the lungs to a considerable extent at the upper part of the sternum; it likewise causes the central tendon of the diaphragm to protrude to a great extent into the abdomen, displacing the liver and stomach. In disease of the heart with enlargement, the lungs are displaced to an extent proportionate to the enlargement, but not at the upper part of the sternum; the liver and stomach, the whole diaphragm, and consequently the basis of both lungs, are considerably lower than in the normal state.

In enlargement of the liver the free play of the lungs and heart is interfered with if the patient lies on the left side, the weight of the liver falling upon the heart interferes with its action, and causes palpitation. In persons affected with ovarian dropsy or ascites, the abdominal viscera, and consequently the diaphragm, are pushed abnormally upwards, and the lower borders of the lungs and heart are unnaturally high.

The same state obtains, but to a more extreme degree, when the stomach or intestines are over distended as in dyspepsia, this state inducing palpitation; or in peritonitis, where the enormous distension often seriously interferes with the action of the heart and lungs. In empyema and its extensive pleuritic effusion the diaphragm is more lowered and the abdominal viscera are more displaced than in any other disease of the lungs.—*Med. Examiner.*—*Provin. Medical Journal*, Aug. 12, 1843.

